# Rasopathy-Associated Mutation Ptpn11^D61Y^ has Age-Dependent Effect on Synaptic Vesicle Recycling

**DOI:** 10.1007/s10571-024-01505-1

**Published:** 2024-11-21

**Authors:** Debarpan Guhathakurta, Franziska Selzam, Aneta Petrušková, Eva-Maria Weiss, Enes Yağız Akdaş, Carolina Montenegro-Venegas, Martin Zenker, Anna Fejtová

**Affiliations:** 1 Department of Psychiatry and Psychotherapy, Universitätsklinikum Erlangen, Friedrich-Alexander-Universität Erlangen-Nürnberg, Erlangen, Germany; 2 RG Presynaptic Plasticity, Leibniz Institute for Neurobiology, Magdeburg, Germany; 3 Institute of Human Genetics, Medical Faculty, University Hospital Magdeburg, Otto Von Guericke University, Magdeburg, Germany; 4 National Institute of Mental Health, Klecany, Czech Republic; 5 Third Faculty of Medicine, Charles University, Prague, Czech Republic; 6 Department of Neurochemistry and Molecular Biology, Leibniz Institute for Neurobiology, Magdeburg, Germany; 7 Present Address: Institute for Pharmacology and Toxicology, Medical Faculty, Otto von Guericke University, Magdeburg, Germany

## Abstract

Rasopathies are genetic disorders often associated with developmental delay and intellectual disability. Noonan syndrome (NS) is one of the most common Rasopathies, caused by mutations in PTPN11 in more than 50% of cases. In mammalian neurons, PTPN11 controls the trafficking of postsynaptic glutamate receptors. This process is disrupted in neurons expressing PTPN11 variants associated with Rasopathies and is thought to contribute to the cognitive impairments in Noonan syndrome. Recent work revealed presynaptic impairments upon expression of RASopathy-linked PTPN11 variants in Drosophila. However, the presynaptic role of PTPN11 has not yet been addressed in mammals. Here, we investigated membrane trafficking of synaptic vesicles in cultured mouse cortical neurons expressing Rasopathy-associated PTPN11^D61Y^ variant. We observed a significantly smaller readily releasable and total recycling pool of synaptic vesicles. The drop in synaptic vesicle release competence was accompanied by a decreased rate of SV retrieval. Interestingly, the presynaptic phenotype was evident in mature (DIV21) but not in immature (DIV12) neurons. Thus, our data reveal importance of balanced PTPN11 activity for normal trafficking of neurotransmitter-filled synaptic vesicles in the presynaptic ending of mature neurons.

**Supplementary Information:**

The online version contains supplementary material available at 10.1007/s10571-024-01505-1.

## Introduction

Rasopathies are a group of genetic disorders caused by germline mutations in genes encoding for components or regulators of the cellular RAS-MAPK signalling cascade, which is important for cellular growth, proliferation and differentiation (Tidyman and Rauen 2009; Seger and Krebs 1995). At the cellular level, RASopathies share an increased activity of cellular RAS signalling. Noonan syndrome (NS) is one of the most common RASopathies. It is characterized by reduced growth, craniofacial abnormalities, congenital heart defects, developmental delay and neurocognitive abnormalities of variable degree (Roberts 1993). Despite common occurrence of neuropsychiatric symptoms in NS it is unclear, how increased PTPN11 activity affects neuronal function. Importantly, the expression of NS-linked mutations in mice led to cognitive impairments (Altmüller et al. 2017; Lee et al. 2014; Ryu et al. 2019). Multiple distinct mutations in PTPN11 can be identified in NS patients (Tartaglia et al. 2003). Neurobiological studies have been performed in mice expressing several PTPN11 variants, including PTPN11^D61G^ and PTPN11^N308D^, which are associated with NS and PTPN11^D61Y^, which occurs as a somatic mutation in humans and leads to juvenile myelomonocytic leukaemia (JMML) (Tartaglia et al. 2003; Lee et al. 2014; Altmüller et al. 2017; Ryu et al. 2019). Biochemically, PTPN11^D61Y^ mutation leads to a more pronounced increase in the basal activity of RAS signalling compared to PTPN11^D61G^ and PTPN11^N308D^ (Tartaglia et al. 2003). Despite their different level of MAPK activation, the expression of RASopathy-linked PTPN variants in brain results in similar behavioural, molecular, cellular, and electrophysiological phenotypes (Lee et al. 2014; Ryu et al. 2019; Altmüller et al. 2017). This indicates a convergent mechanism by which irregular PTPN11 activity affects neuronal function and justifies the investigation of neuronal effect of PTPN11^D61Y^ that primarily causes JMML.

The studies that have investigated the cellular and molecular mechanism underlying the neuronal defects in NS mostly focussed on postsynaptic glutamate receptors and revealed their aberrant trafficking and surface expression in mammalian neurons expressing the PTPN11^D61G^ and PTPN11^D61Y^ variants (Ryu et al. 2020; Levy et al. 2018; Altmüller et al. 2017). In contrast to that, limited number of studies have addressed the role of PTPN11 in the presynaptic terminal. Drosophila PTPN11 homologue, corkscrew (csw), was shown to limit glutamatergic neurotransmission in the fly neuromuscular junction (NMJs) via presynaptic and MAPK-signalling-dependent mechanisms (Leahy et al. 2023). Interestingly, both csw loss-of-function (LoF) and RASopathy-related gain-of-function (GoF) mutants that disrupt the autoinhibition of csw phosphatase activity increased neurotransmission by changing release characteristics of synaptic vesicles (SVs) (Leahy et al. 2023). SVs can be functionally classified into ready releasable pool (RRP), recycling pool and resting pool (Rizzoli and Betz 2005). The vesicles of RRP are available for immediate evoked release and can be morphologically identified as docked vesicles at the presynaptic membrane. The RRP is rapidly replenished from recycling pool, while vesicles of resting pool are recruited upon high-frequency stimulation. In csw LoF and GoF NMJs, physiological and morphological analysis confirmed increased RRP, unchanged synaptic release probability and differences in the presynaptic short-term plasticity (Leahy et al. 2023, 2024). Interestingly, the deletion of Drosophila synapsin (dSyn) or pharmacological or genetic interference with MAPK-signalling normalised the elevated neurotransmission in csw LoF and GoF NMJs. Synapsin (Syn) is a SV-associated phosphoprotein that promotes clustering of SVs (Cesca et al. 2010). Phosphorylation of synapsin by MAPK promotes dissociation of SVs from the cluster and promotes their docking increasing the RRP (Cesca et al. 2010). Thus, the studies in flies indicated that fly homologue of PTPN11 controls SV pools and neurotransmission via limiting the MAPK-dependent phosphorylation of SV-associated protein synapsin (Leahy et al. 2024). Cultured neurons from mice expressing the PTPN11^D61Y^ variant had normal density of excitatory and inhibitory synapses as well as unchanged proportion of presynaptically active synapses, capable of depolarization-induced neurotransmitter release (Altmüller et al. 2017). However, presynaptic characteristics in neurons expressing NS-linked PTPN11 variants were not yet specifically addressed. Previous work also established developmental stage-specific effect of RASopathy mutations in mammalian neurons. Specifically, the neurons expressing the PTPN11^D61Y^ variant also showed elevated neuronal network activity during early network development, which was normalised later indicating early functional defects and development of compensatory network adaptations (Weiss et al. 2024).

Motivated by recent data obtained in fly system and taking into account the previously described developmental effects, we decided to analyse SV pools in juvenile and mature mammalian neurons expressing the PTPN11^D61Y^ variant. We employed state of art live-imaging of a genetically engineered pH sensor localised to the lumen of SV to monitor their SV fusion and retrieval, which allows to quantify the SV pools as well as kinetics of SV retrieval at level of individual synapses. We also assessed the effects of this variant on the membrane trafficking of postsynaptic GluA receptors in mature neurons.

## Materials and Methods

### Animals and Genotyping

Conditional mice with forebrain specific expression of Ptpn11^D61Y^ used in this study were described and characterized previously (Altmüller et al. 2017). They were obtained from crossings of heterozygous conditional Ptpn11^LSLD61Y/WT^ mice (B6.129S6-Ptpn11tm1Toa/Mmjax, Jackson Laboratories, RRID# MMRRC_032103-JAX) with homozygous Emx1-cre mice (B6.129S2-Emx1tm1(cre)Krj/J, Jackson Laboratories, RRID# IMSR_JAX:005628). Ptpn11^LSLD61Y^ allele contains a LOX-STOP-LOX cassette before the exon containing the RASopathy mutation (Chan et al. 2009). Emx1-cre mice express cre recombinase under control of endogenous EMX1 promoter that drives its expression in forebrain excitatory neurons and glia from embryonic day 10.5 on (Gorski et al. 2002). Ptpn11^WT/WT^ x Emx1-cre^cre/WT^ are referred to as control and mice bearing the mutated allele Ptpn11^LSLD61Y/WT^ x Emx1-cre ^cre/WT^ are referred to as Ptpn11^D61Y^. Both strains were backcrossed into the C57BL/6N background for more than 5 generations. Mice were bred in Franz-Penzoldt-Zentrum (FPZ) in Erlangen and experiments were carried out in accordance with local regulations and following the European Directive 2010/63/EU and registered under local Az:: TS12/2016 and TS2/2023.

### Mice Cortical Culture Preparation

New born (P0-P1) Ptpn11^D61Y^ and control mice were used for preparation of cortical culture as described previously (Guhathakurta et al. 2024). Briefly, the cortical hemispheres freed from soft cranial bones and meninges were incubated at 37 °C for 10 min in a solution containing 0.127 U/ml papain (#LK003176, Worthington Biochemical Corporation), 1 mg/ml dispase II (#04942078001, Roche), 0.1 mg/ml DNase I (#LS002139, Worthington) and 12.4 mM MgSO4. Then, tissues were mechanically triturated. This cycle of incubation and trituration was repeated three times to obtain a homogenous cell suspension. The cell suspension was filtered through 70 μm nylon cell strainers, centrifuged at 120 g for 5 min and resuspended in Neurobasal A media (#12349–015) supplemented with 1% (v:v) GlutaMAX™ (#35050–038), 1 mM sodium pyruvate (#11360–070), 1% (v:v) Antibiotic/Antimycotic and 2% (v:v) B27 (all from Thermo Fisher Scientific). ø18 mm Menzel glass coverslips (#6311342, VWR international) were coated with 0.5 mg/ml Poly-L-Lysine (PLL, #P1524, Sigma-Aldrich). 200.000 cells in total volume of 100 µl were seeded on the coverslips and left to attach for 1 h at 37 °C, 5%CO_2_. Then, coverslips were transferred to 12-well plates containing 1 ml supplemented Neurobasal A media and maintained in the humidified incubator at 37 °C with 5% CO2 till the experimental date, while 30% of media was replaced once per week. Cortical neurons from the control or Ptpn11^D61Y^ were maintained in the same 12-well plate to ensure uniform conditions of handling.

### Lentiviral Particle Production and Transduction

The SypmOr sensor used for live-imaging in this study has been reported previously (Egashira et al. 2015). The probe was expressed in cortical neurons using lentiviral vectors prepared and applied as described previously (Guhathakurta et al. 2022). Briefly, lentiviral particles were produced in HEK293T cells (ATCC) upon transfection with FUW-based SypmOr construct, psPAX2 (#12260, Addgene) and pVSVG (#8454, Addgene) vector at 1.64:0.72:1.3 (in pM) concentrations (in total 10 µg), respectively, using 30 µl FuGENE® HD (Promega) and the manufacturer´s protocol. Transfection media were replaced with supplemented Neurobasal A media 16 h after transfection. 24 h later, lentiviral particles were collected and 100 µl of collected media was used for transduction of day in vitro (DIV) 2 neuronal cultures from control and Ptpn11^D61Y^ mice.

### SypmOr Live-Imaging

Live-imaging was done using motorised Ti Eclipse microscope equipped with 60X/NA1.2 water-immersion objective (CFI Plan Apo VC, Nikon), iXon EM + 885 EMCCD Andor camera (Andor Technology) and Omicron LedHUB illumination (Omicron-laserage Laserprodukte GmbH) and controlled by VisiView software (Visitron System GmbH). At the day of imaging experiment, coverslips with neurons were briefly washed with TB and mounted in a imaging chamber (#RC-49MFSH, Warner Instrument, Hamden, Connecticut, USA) containing platinum electrodes placed 1 cm apart. Live-imaging was performed in TB containing 10 μM CNQX, 50 μM APV to block spontaneous network activity at 30 ± 2 °C. Bafilomycin A1(1 μM), a blocker of the vesicular proton pump preventing the re-acidification of endocytosed SVs, was added for imaging of SV pools to visualise all vesicles that underwent exocytosis during experiment duration. SypmOr response was acquired using Cy3 filter-set (in mm: excitor 543/22 and emitter 593/40). Cultures that did not respond to the stimulation were discarded. This happened rarely and affected entire batch prepared at same day. Image acquisition for SV pool quantification and for SV retrieval was carried out in 2 * 2 binning mode at 1 or 3.33 Hz, respectively. SV pools imaging was adapted from (Guhathakurta et al. 2024, 2022). Briefly, after 15 secs of baseline recording, 40 APs at 20 Hz and 900 APs at 20 Hz were delivered using A385 stimulus isolator (World Precision Instruments) connected to a stimulus generator (#STG-4008, 240 Multi-Channel Systems) to release the readily releasable pool (RRP) and total recycling pool (TRP) of SVs, respectively. Then, 60 mM NH_4_Cl was applied to achieve alkalization and unquenching of all SypmOr expressing vesicles. For imaging exo-endocytosis of SVs, after 10 secs of baseline recording 40AP at 20 Hz was delivered to release RRP and fluorescence recovery was monitored for an additional 45 secs.

### SypmOr Live-Imaging Analysis

Data processing and analysis of SV pools imaging were done as described previously (Guhathakurta et al. 2024). Briefly, after baseline subtraction, maximal intensity z-projection was created from 45 frames during 900 AP stimulation and an automated ImageJ plugin SynQuant for synaptic puncta detecting (Wang et al. 2020) was utilized to define 150–200 ROIs around responding synaptic puncta according to users application manual. To obtain values for quantification, ROIs defined in the first step were applied to all frames, and mean fluorescence intensities (F) in ROIs were normalized by standard min to max feature scaling formula (F-Fmin)/(Fmax-Fmin), where Fmin corresponds to average intensity of baseline frames [12 frames corresponding to time interval between 3 and 15 s from the start of recording (baseline)] and Fmax corresponds to the NH_4_Cl response. Only samples where fluorescent intensity change was stable upon 40 and 900 AP stimulations and ≤ 80% of NH_4_Cl-evoked fluorescence were included for the analysis. The relative fractions of RRP (measured from 10 frames corresponding to time interval between 40 and 50 s) and TRP (10 frames corresponding to time interval between 150 and 160 s) of SVs were normalised to the maximum fluorescence recorded upon NH_4_Cl alkalization (Fmax). Similarly, for quantification of exo-endocytosis of RRP, a maximal z-projection of 10 frames during 40 AP stimulation was created and ROIs containing responding synaptic puncta were defined with SynQuant. Fluorescence intensities were read out and each value was normalized to the maximum F value reached upon 40 AP in order to obtain a trace. Every 5 frames from the acquired image stack were averaged to obtain the represented traces. F values from the recovery part of the trace were fitted onto a non-linear regression fit model (one-phase decay) and half-time of recovery was calculated by fitting the fluorescence decay to a non-linear regression fit model using GraphPad prism.

### Staining of Surface Glutamate Receptors

For staining of surface fraction of GluA receptors, mouse antibody against panGluA (Oyster550-labelled, 1:500, # 182411C3, Synaptic Systems) was applied in Tyrode´s buffer (TB; in mM: 119 NaCl, 2.5 KCl, 2 CaCl2, 2 MgCl2, 30 glucose, 25 HEPES; pH = 7.4) to DIV21 cortical neurons for 20 min at 37 °C. This antibody recognizes extracellular epitope of GluA1, GluA2 and GluA3 receptors. Then, cells were with 4% (w:v) paraformaldehyde (PFA) in PBS for 4 min at RT, blocked and permeabilized with PBS solution containing 10% (V:V) FCS and 0.1% (w:v) glycine and 0.3% (v:v) TritonX-100 for 40 min. Primary rabbit antibody against Shank2 (1:1000, #162204, Synaptic Systems, K.O. validated), guinea pig antibody against Synapsin 1,2 (1:1000, #106104, Synaptic System, K.O. validated) and secondary antibody (anti-rabbit Alexa 488 (1:1000, #711545152, Jackson Immunoresearch) and anti-guinea pig Cy5 (1:1000, # 706175148, Jackson Immunoresearch) were applied in PBS containing 3% (v:v) FCS overnight at 4 °C and for 1 h at RT, respectively. Coverslips were then mounted on glass slides with Fluoroshield™ (#F6182, Sigma-Aldrich). All experimental conditions investigated in each experiment were processed in parallel with identical antibodies, solutions, and other chemicals.

### Quantitative Image Acquisition and Analysis

16-bit images of stainings were acquired using microscope described above. Identical camera and illumination settings were applied during image acquisition for coverslips of all experimental conditions imaged on the same day. An average of 10 visual fields was imaged from each coverslip. For analyses of GluA and Syn1, 2 staining 16-bit images were analysed using in-house MATLAB routine SynEval (https://github.com/EvaMWe/Synapse-quantification). This includes the identification of synaptic puncta using a segmentation algorithm that follows an iterative, watershed based approach and measurement of according fluorescence intensity (FI) in all channels (Guhathakurta et al. 2022). Labelling of Syn1/2 was used to identify synaptic puncta (i.e. to create a segmentation mask) and Shank2 to identify excitatory synapses.

### Statistics

An a priori calculation of sample size was not performed before conducting the study. Instead, sample size for all experiments was set according to previous publications, where the comparable parameters were assessed using the same methodology (Montenegro-Venegas et al. 2022; Guhathakurta et al. 2024). Statistical analyses were performed using GraphPad Prism 9.5.1 (GraphPad Software, San Diego, California, USA). All data points for quantitative analyses are depicted as scatter plot with bar showing the mean and whiskers indicating the SEM. Group size, i.e. number of independently treated and processed coverslips, and number of animals used as source of cells, is indicated for all analyses in the figures and in the Supplementary Tables 1–3. Assumptions of statistical tests (i.e. normal distribution and distribution of variance) were tested using the D´Agostino-Pearson test for each data set, and appropriate tests were then applied accordingly. For normally distributed samples, significance was assessed using the two-tailed unpaired Student`s t test and marked as * for *p* < 0.05, ** for *p* < 0.005 and *** for *p* < 0.0005 in all plots.

### Reproducibility and Variability

To ensure the reproducibility and reliability of our experiments, neuronal cultures were prepared from multiple independently processed animals, namely for immunocytochemistry experiments 3 per genotype and for imaging 7–10 per genotype. By incorporating these biological replicates, we effectively accounted for physiological variability, thereby increasing the robustness of our findings and confirming that observed effects are consistent and scientifically valid. Coverslips with neurons from control or Ptpn11^D61Y^ pups were grown in one 12-well plate to reduce variability and bias. Imaging, staining and analysis were done without knowing the respective genotype. Additionally, all experimental conditions were rigorously controlled: each condition was processed concurrently using identical antibodies, solutions and reagents to minimize variability and enhance the reproducibility of the results.

## Results

### Mature Ptpn11^D61Y^ Neurons Display Changes in SV Recycling

**Fig. 1:**
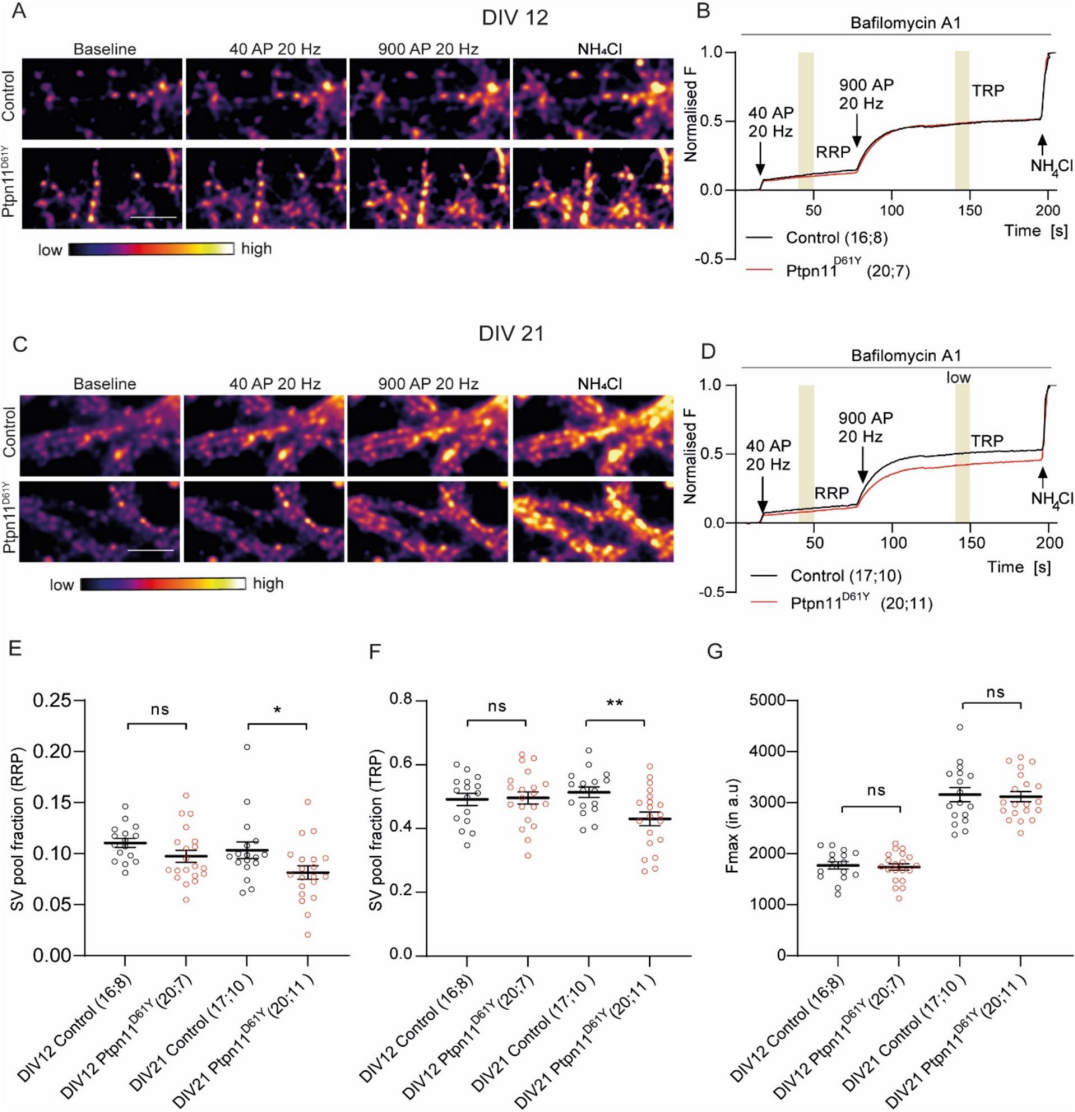
Developmental effect of Ptpn11^D61Y^ mutation on SV pools. **A**, **C** Representative colour gradient images show SypmOr fluorescence at baseline, upon stimulation with 40 or 900 APs at 20 Hz and upon application of NH_4_Cl in DIV12 and DIV21 cortical control and Ptpn11^D61Y^ neurons. **B**, **D** Average traces show SypmOr fluorescence in DIV12, DIV21 control and Ptpn11^D61Y^ neurons, respectively. **E** Quantification of RRP and **F** TRP fraction of SVs from traces. **G** Quantification of maximal SypmOr fluorescence. Significance was assessed using unpaired t test and is indicated as **p < 0.005, *p ≤ 0.05. Sample size is indicated in brackets and shows the independent number of independently processed coverslips or animals used. Scale bar is 5 µm

In this study, we aimed to investigate the effect of Ptpn11^D61Y^ mutation on dynamics of SVs within presynaptic ending. For this purpose, we monitored SV release and recycling in cultured neurons using a pH-sensitive probe called synaptophysin-mOrange [SypmOr (Egashira et al. 2015)]. SypmOr contains a pH-sensitive RFP variant inserted within the lumenal domain of SV protein synaptophysin. Its fluorescence increases during SV fusion when it gets exposed to neutral pH of extracellular solution and decreases again upon compensatory endocytosis and re-acidification of SVs. We applied an established stimulation protocol consisting of 40 action potential (AP) at 20 Hz to drive the fusion of docked SVs (corresponding to the readily releasable pool of SVs, RRP) followed by application of 900 AP at 20 Hz to release all release‐competent SVs (TRP) (Burrone et al. 2006). We analysed cells cultured for 12 or 21 days (Fig. 1A–D). These time points were selected to cover the span of time before and after the full functional presynaptic maturation (Wierenga et al. 2006). The imaging revealed no changes in RRP and TRP of SVs in DIV12 Ptpn11^D61Y^ neurons compared to controls (Fig. 1A, B, E, F; Supplementary Table 1A). In contrast to that in DIV21 Ptpn11^D61Y^ neurons, RRP and TRP of SVs were reduced by about 20% as compared to the controls (Fig. 1C–F, Supplementary Table 1A). The expression of SypmOr sensor did not differ between genotypes at both time points, indicating no changes in abundance of SVs between genotypes (Fig. 1G, Supplementary Table 1B). These experiments revealed a developmental decline in size of RRP and TRP in neurons expressing overactive Ptpn11^D61Y^ variant.

### Ptpn11^D61Y^ Mutation Leads to Delayed Retrieval of RRP of SVs

**Fig. 2:**
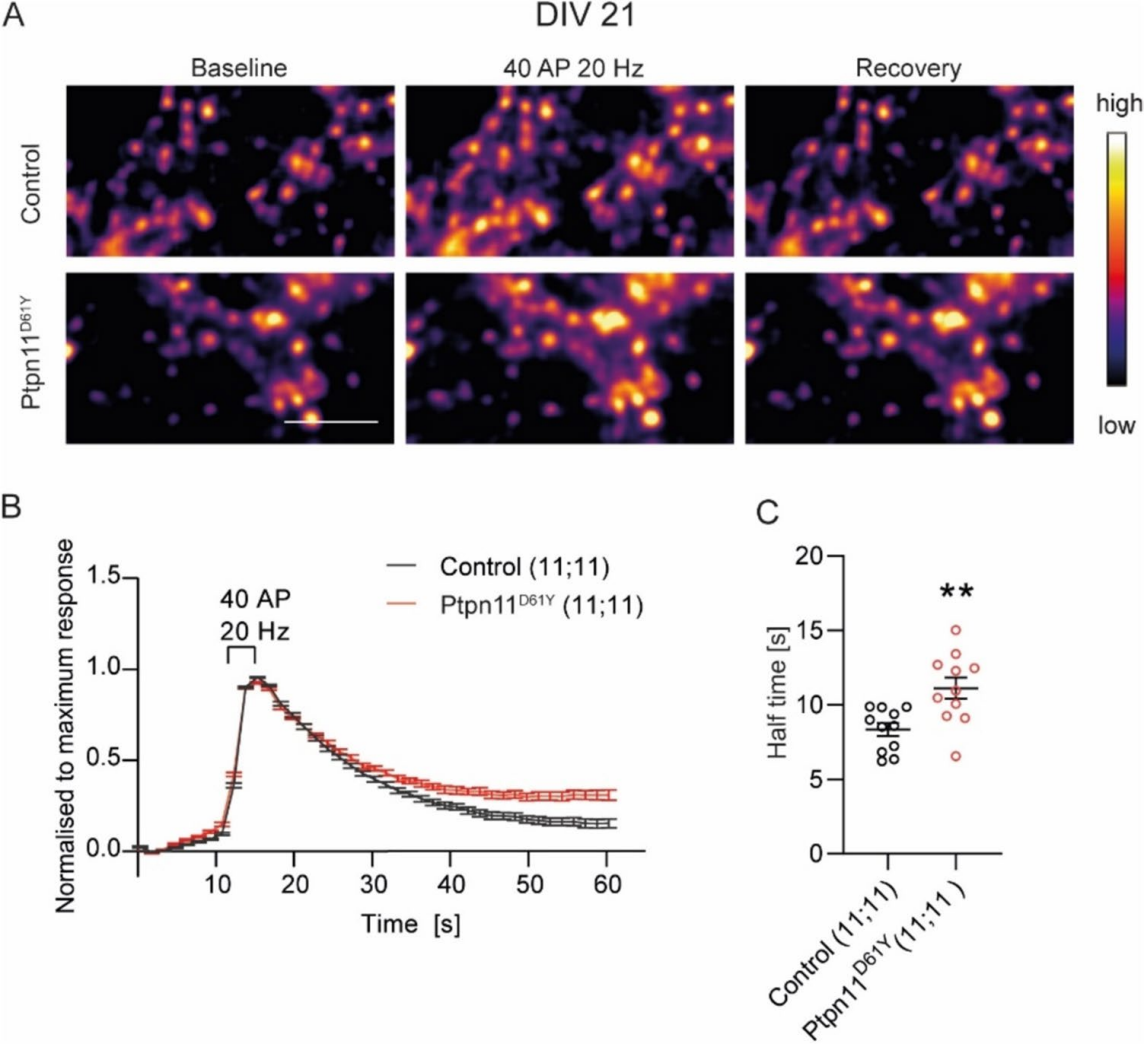
RRP retrieval is delayed in Ptpn11^D61Y^ neurons. **A** Representative colour gradient images of control and Ptpn11^D61Y^ cortical neurons expressing SypmOr during baseline, upon stimulation with 40 APs at 20 Hz, and during recovery. **B** Averaged SypmOr-fluorescence traces normalized to maximum response showing kinetics of RRP retrieval in Ptpn11^D61Y^ and control neurons. **C** Quantification of estimated half-time of RRP retrieval. Significance was tested using unpaired t test and is indicated as **p < 0.005. Numbers in brackets show the number of independently processed and analysed cultures and animals used. Scale bar is 5 µm

Dynamics of SV retrieval determine rate and routes of SV recycling (Wu 2004). To test whether defects in SV retrieval underlie the reduced recycling of SVs in Ptpn11^D61Y^ neurons, we assessed the kinetics of SypmOr response evoked by 40 AP at 20 Hz in control and Ptpn11^D61Y^ neurons (Fig. 2A). This stimulus mobilizes the docked SVs (i.e. RRP) (Stevens and Williams 2007). The rise time of the evoked SypmOr response, reflecting the evoked exocytosis, was indistinguishable between Ptpn11^D61Y^ and control neurons, but, decay reflecting the SV retrieval was significantly slower as assessed from the calculated fluorescence decay half-time in Ptpn11^D61Y^ neurons (Fig. 2B,C, Supplementary Table 2). The slower decay of SypmOr fluorescence speaks for slower endocytosis of SVs. However, we cannot exclude contribution of slower re-acidification of retrieved SVs. Both these reasons would argue for changes in rates and/or routes of endocytosis, which could contribute to the observed decrease in the SV pool sizes in Ptpn11^D61Y^ neurons.

### Ptpn11^D61Y^ Mutation Leads to Increased Surface Abundance of GluA Receptors

**Fig. 3:**
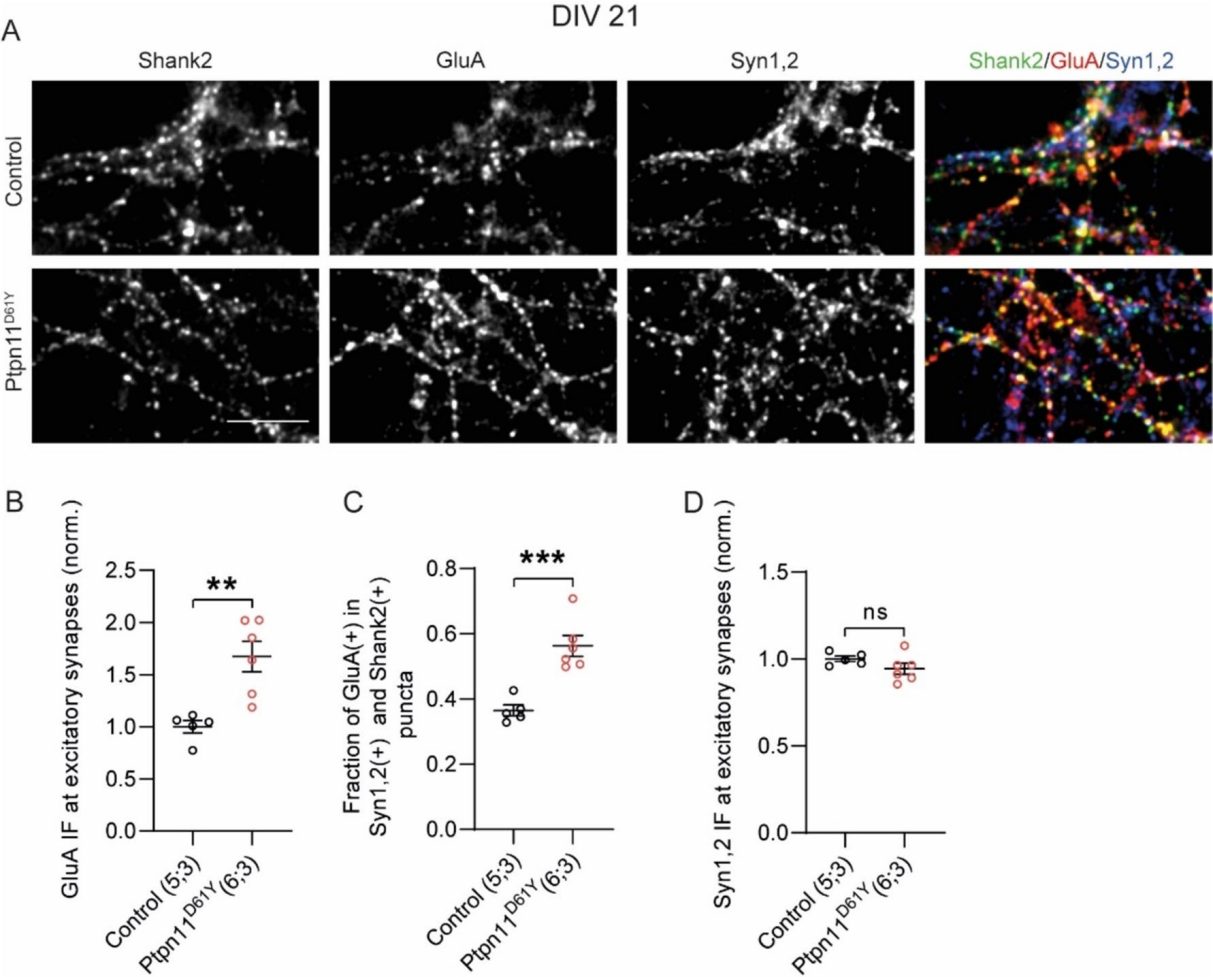
Synaptic surface expression of GluA receptors is increased in Ptpn11^D61Y^ neurons. **A** Representative images showing live-labelling of synaptic GluA receptors in cortical neurons from control and Ptpn11^D61Y^ mice. Excitatory synapses were labelled by co-staining for Syn1,2 and Shank2. **B** Quantification IF intensity of staining for synaptic GluA, **C** fraction of GluA positive puncta in the population of excitatory synapses and **D** IF intensity of staining for synaptic Syn1,2. Significance was assessed using unpaired t test and is indicated as ***p < 0.0005, **p < 0.005. Sample size is indicated in brackets and corresponds to the number of independently processed and analysed coverslips and animals they originated from. Scale bar is 5 µm

Ptpn11 was previously shown to control surface expression of postsynaptic glutamate receptors (Zhang et al. 2016; Lu et al. 2020; Altmüller et al. 2017; Ryu et al. 2019; Oh et al. 2017). Previous work investigating the surface expression of glutamate receptors in neurons expressing Ptpn11^D61Y^ variant showed relatively higher surface abundance and lower endocytosis of GluA compared to controls at DIV14 (Altmüller et al. 2017). Seeing the effect of age on presynaptic SV recycling, we decided to quantify the surface abundance of AMPAR also in DIV21 control and Ptpn11^D61Y^ cortical neurons. Therefore, we performed live labelling of GluA receptors with a fluorophore-tagged antibody recognizing the extracellular epitope of GluAs in DIV21 neurons (Fig. 3A). The synaptic GluA IF increase by nearly 70% in DIV21 Ptpn11^D61Y^ neurons compared to controls (Fig. 3B, Supplementary Table 3). In cortical neurons, a significant portion of excitatory synapses are silent, because they do not express GluA receptors on their surface being unable to response to evoked glutamate release (Kerchner and Nicoll 2008). Our analyses revealed that significantly higher proportion of excitatory synapses identified by co-labelling with antibody against Syn1/2 and Shank2 showed surface immunoreactivity for GluAs i.e. were postsynaptically active in Ptpn11^D61Y^ neurons compared to control neurons (Fig. 3C; Supplementary Table 3). Finally, we also quantified abundance of synaptic vesicle-associated protein Syn1/2, which was shown to be regulated in synapses of flies expressing RASopathy-linked PTPN11 variants (Leahy et al. 2024). In contrast to findings in flies, we did not observe any changes in the abundance of Syn1/2 between genotypes (Fig. 3D). Thus, similarly as described in DIV14 neurons from the same animal model in previous studies, we observed an increased surface expression of AMPA receptors and increased number of synapses expressing GluA on their surface in mature (DIV21) neurons expressing RASopathy-linked Ptpn11^D61Y^ variant.

## Discussion

In the present work, we investigated functional SV pools and SV recycling at excitatory synapses of cultured primary cortical neurons prepared from mice expressing RASopathy-associated Ptpn11^D61Y^ variant. We observed no changes in RRP in DIV12 cells and a significantly reduced size of RRP and TRP in DIV21 neurons. It has been suggested that presynaptic activity correlates with activity of neuronal RAS-MAPK signalling (Sweatt 2004). Increased synaptic release probability and larger RRP were also reported in juvenile (21 days old) and adult (3–6 months) mice expressing the constitutively active HRAS^G12V^, which leads to Costello syndrome, a specific RASopathy (Seeger et al. 2004; Kushner et al. 2005).We have observed that the SV pools were unchanged in DIV12 Ptpn11^D61Y^ neurons, in which the downstream effectors of RAS signalling were more active (Altmüller et al. 2017). Thus, the changes in SV pools do not directly correlate with the increased RAS-MAPK signalling in mammalian neurons expressing the Ptpn11^D61Y^ variant. It is possible that distinct RASopathy-linked mutations specifically affect presynaptic properties.

In contrast to our finding, expression of RASopathy-linked mutations in Drosophila PTPN11 homologue csw leading to an increased pERK activity also resulted in an increase in the size of RRP as assessed by electrophysiological recordings as well as by immunohistochemistry of NMJ synaptic boutons of Drosophila larvae (Leahy et al. 2024, 2023). Moreover, while in Drosophila the synaptic abundance of phosphoprotein Syn was elevated in NMJ synaptic boutons in animals expressing RASopathy-linked PTPN11 variants, we did not observe any changes (Leahy et al. 2024). This is likely due to usage of different experimental systems. While Drosophila NMJ represents a simple connection between glutamatergic motoneuron and muscle fibre, rodent neuronal cultures form complex networks of excitatory and inhibitory neurons. Indeed, previous data revealed progressive changes in the neural network activity in neurons expressing Ptpn11^D61Y^ variant (Weiss et al. 2024). In this study, longitudinal recordings of neurons grown on multielectrode arrays (MEAs) revealed elevated spiking and bursting in DIV12-15 neurons expressing Ptpn11^D61Y^. This heightened activity progressively normalized and was not significantly different from controls at DIV21 (Weiss et al. 2024). Interestingly, despite this apparently normal network activity, the evoked responses and excitation/inhibition balance were disturbed in these networks, indicating development of adaptive mechanisms (Weiss et al. 2024).

Neuronal networks have been shown to employ mechanisms of homeostatic synaptic plasticity to stabilise their firing rate. Under conditions of increased neuronal firing, intrinsic neuronal excitability and synaptic transmission strengths decrease, whereas both parameters increase upon chronic withdrawal or reduction of neuronal firing (Burrone et al. 2002; Turrigiano et al. 1998). Regulation of SV pool size is one of the mechanisms that modulate neurotransmitter release and act to stabilise firing rates in neuronal networks (Kim and Ryan 2010; Murthy et al. 2001; Lazarevic et al. 2011). Based on our observations, we propose that the decrease in recycling SV pools that we observed in DIV21 but not in DIV12 Ptpn11^D61Y^ neurons likely reflects a homeostatic adaptation to the increased firing rate described during early network development in our recent study (Weiss et al. 2024). It has been shown that homeostatic scaling also influences kinetics of SV retrieval (Kim and Ryan 2010). Thus, slower retrieval of SVs in Ptpn11^D61Y^ neurons described in this study can also originate from homeostatic adaption to increased network activity and further support the hypothesis that an increased network activity drives the homeostatic adaptation of presynaptic properties. The appearance of the homeostatic decrease in RRP and TRP in DIV21 but not in DIV12 is also consistent with the temporal regulation of the expression locus of homeostatic synaptic plasticity. Wierenga and colleagues showed that neurons cultured for ≤ 15 days express only postsynaptic homeostatic plasticity, whereas neurons cultured for ≥ 18 days show a coordinated pre- and postsynaptic homeostatic response (Wierenga et al. 2006).

The present study also confirmed an increased surface expression of AMPA receptors in DIV21 Ptpn11^D61Y^ neurons. This is consistent with previous reports of increased surface abundance of AMPAR in a related NS mouse model expressing the Ptpn11^D61G^ variant (Lee et al. 2014; Zhou et al. 2022). This effect is due to the direct effect of increased RAS signalling leading to increased phosphorylation events that promote the trafficking and stabilization of AMPARs on the synaptic surface (Zhou et al. 2022; Zhang et al. 2016). Interestingly, we observed an increased proportion of “active” excitatory synapses with surface AMPAR expression in DIV21 Ptpn11^D61Y^ neurons, which was not observed in the same experimental model at an earlier time point (DIV14) (Altmüller et al. 2017). This observation is consistent with a study using neurons expressing the NS-associated Ptpn11^D61G^ variant, where a very similar developmental stage-dependent regulation of AMPAR surface expression was reported (Oh et al. 2017). Previous studies demonstrated that expression of Ptpn11^D61Y^ variant also hampered induction of inactivity-induced homeostatic downscaling, which is normally mediated by endocytosis of AMPARs leading to reduction in their surface expression (Lu et al. 2020). It would be interesting to investigate, whether increased PTPN11 activity also interferes with inactivity-induced elevation of RRP and TRP.

Taken together, this study shows normal SV recycling in DIV12 but significantly reduced sizes of RRP and TRP in DIV21 neurons, which was accompanied by a significant decrease in rate of SV retrieval. Since hyperactive Ras signalling has been associated with enhanced neurotransmitter release and synaptic plasticity (Sweatt 2004), the observed changes in SV pools are unlikely to be a direct effect of increased RAS signalling and likely represent a homeostatic response to the increased network activity balancing the high firing rate in Ptpn11^D61Y^ neurons. In contrast, the changes in AMPA receptor abundance were more consistent with a direct effect of the constitutively active PTPN11 variant on AMPA receptor surface expression. Thus, the expression of RASopathy-related PTPN11 variants affects neuronal transmission not only directly through changes in the intracellular signalling but also indirectly inducing maladaptive functional changes in neuronal networks. This should be taken in account while developing and testing new therapeutic approaches for these diseases.

## Availability of Code, Data, and Materials

Data were obtained according to the material section. The datasets during and/or analysed during the current study are available from the corresponding author upon reasonable request.

## Supplementary Information

Below is the link to the electronic supplementary material.

Supplementary file1 (DOCX 18 KB)
