# Supplementary material for: Rasopathy-Associated Mutation Ptpn11D61Y has Age-Dependent Effect on Synaptic Vesicle Recycling
Source: Cell Mol Neurobiol. 2024 Nov 21;44:77. doi: 10.1007/s10571-024-01505-1 (PMC11582327; doi:10.1007/s10571-024-01505-1)
Supplement: Supplementary file 1 — Supplementary file1 (DOCX 18 KB) [file 10571_2024_1505_MOESM1_ESM.docx]

Supplementary table 1A: Synaptic vesicle pools

| Age of neurons | Sample size (Number of coverslips; number of animals) | Normality and Lognormality Test | SV pool fractions | Control | | Ptpn11^D61Y^ | | Statistical test details and parameters | | | |
| --- | --- | --- | --- | --- | --- | --- | --- | --- | --- | --- | --- |
|  |  |  |  | Passed normality test? | Mean±  SEM | Passed normality test? | Mean±  SEM | Test type | P value and significance | t,df | F, DFn, Dfd |
| DIV 12 | Control –16;8  Ptpn11^D61Y^- 20;7 | D'Agostino & Pearson test | RRP | yes | 0.11 ± 0.004 | yes | 0.10 ± 0.006 | Two-tailed Student`s t-test, unpaired | *p = 0.1104*  *(ns)* | 1.689,34 | 2.274,19,15 |
|  |  | D'Agostino & Pearson test | TRP | yes | 0.49 ± 0.019 | yes | 0.50 ± 0.019 |  | *p = 0.8669*  *(ns)* | 0.168,34 | 1.235,19,15 |
| DIV 21 | Control –17;10  Ptpn11^D61Y^-20;11 | D'Agostino & Pearson test | RRP | No | 0.10 ± 0.008 | yes | 0.08 ± 0.007 | Two-tailed Mann-Whitney test | *p = 0.0281 (*)* | *N.A* | *N.A* |
|  |  | D'Agostino & Pearson test | TRP | yes | 0.51 ± 0.016 | yes | 0.43 ± 0.021 | Two-tailed Student`s t-test, unpaired | *p = 0.0047 (**)* | 3.019,35 | 2.051,19,16 |

Supplementary table 1B: Maximum SypmOr fluorescence

| Age of neurons | Sample size (Number of coverslips; number of animals) | Normality and Lognormality Test | Control | | Ptpn11^D61Y^ | | Statistical test details and parameters | | | |
| --- | --- | --- | --- | --- | --- | --- | --- | --- | --- | --- |
|  |  |  | Passed normality test? | Mean±  SEM | Passed normality test? | Mean±  SEM | Test type | P value and significance | t,df | F, DFn, Dfd |
| DIV 12 | Control –16;8  Ptpn11^D61Y^- 20;7 | D'Agostino & Pearson test | yes | 1773 ± 72.020 | yes | 1740 ±  63.970 | Two-tailed Student`s t-test, unpaired | *p = 0.7345 (ns)* | 0.345,34 | 1.014, 15, 19 |
| DIV 21 | Control –17;10  Ptpn11^D61Y^-20;11 | D'Agostino & Pearson test | yes | 3159 ± 138.100 | yes | 3119 ± 99.530 | Two-tailed Student`s t-test, unpaired | *p = 0.8121 (ns)* | 0.240,35 | 1.636,16,19 |

Supplementary table 2: Half-time of SV retrieval

| Age of neurons | Sample size (Number of coverslips; number of animals) | Normality and Lognormality Test | Control | | Ptpn11^D61Y^ | | Statistical test  details and parameters | | | | |
| --- | --- | --- | --- | --- | --- | --- | --- | --- | --- | --- | --- |
|  |  |  | Passed normality test? | Mean±  SEM | Passed normality test? | Mean±  SEM | Test type | | P value and significance | t,df | F, DFn, Dfd |
| DIV21 | Control – 11;11  Ptpn11^D61Y^- 11;11 | D'Agostino & Pearson test | yes | 8.34 ± 0.432 | yes | 11.12 ± 0.714 | Two-tailed Student`s t-test, unpaired | *p = 0.0034 (**)* | | 3.324;20 | 2.726,10,10 |

Supplementary table 3. GluA surface labeling and Syn1,2 IF

| GluA Immunofluorescence at excitatory synapses | | | | | | | | | | |
| --- | --- | --- | --- | --- | --- | --- | --- | --- | --- | --- |
| Age of neurons | Sample size (Number of coverslips; number of animals) | Normality and Lognormality Test | Control | | Ptpn11^D61Y^ | | Statistical test  details and parameters | | | |
|  |  |  | Passed normality test? | Mean±  SEM | Passed normality test? | Mean±  SEM | Test type | P value and significance | t,df | F, DFn, Dfd |
| DIV21 | Control – 5;3  Ptpn11^D61Y^- 6;3 | D'Agostino & Pearson test | yes | 1.00 ± 0.060 | yes | 1.67 ± 0.146 | Two-tailed Student`s t-test, unpaired | *p = 0.0033*  *(**)* | 3.968,9 | 7.311,5,4 |
| GluA positive fraction at excitatory synapses | | | | | | | | | | |
| DIV21 | Control – 5;3  Ptpn11^D61Y^- 6;3 | D'Agostino & Pearson test | yes | 0.37 ± 0.017 | yes | 0.56 ± 0.032 | Two-tailed Student`s t-test, unpaired | *p = 0.0006*  *(***)* | 5.158,9 | 4.379,5,4 |
| Syn1, 2 immunofluorescence at excitatory synapses | | | | | | | | | | |
| DIV21 | Control – 5;3  Ptpn11^D61Y^- 6;3 | D'Agostino & Pearson test | yes | 1.00 ± 0.016 | yes | 0.94 ± 0.032 | Two-tailed Student`s t-test, unpaired | *p = 0.1712 (ns)* | 1.487,9 | 4.724,5,4 |
